# Homotypical and Heterotypical Intergenerational Continuity of Child Maltreatment: Evidence from a Cohort of Families Involved with Child Protection Services

**DOI:** 10.3390/ijerph20054151

**Published:** 2023-02-25

**Authors:** Rachel Langevin, Audrey Kern, Tonino Esposito, Sonia Hélie

**Affiliations:** 1 Department of Educational and Counselling Psychology, McGill University, 3700 Rue McTavish, Montréal, QC H3A 1Y2, Canada; 2 School of Social Work, Université de Montréal, 3150 Rue Jean-Brillant, Montréal, QC H3T 1N8, Canada; 3 Institut Universitaire Jeunes en Difficulté, CIUSSS du Centre-Sud-de-l’Ile-de-Montréal, 1001 Boulevard de Maisonneuve Est, Montréal, QC H2L 4P9, Canada

**Keywords:** child maltreatment, child protection data, intergenerational continuity, multivariate models

## Abstract

Child maltreatment (CM) in one generation can predict CM in the next generation, a concept known as intergenerational continuity. Yet, the form taken by the intergenerational continuity of CM remains unclear and fathers are mostly absent from this literature. This longitudinal study aimed to document patterns of intergenerational continuity of substantiated CM, on the maternal and paternal sides, by examining the presence of: homotypical CM, which is the same type of CM in both generations; and heterotypical CM, which is different CM types in both generations. The study included all children substantiated for CM with the Centre Jeunesse de Montréal between 1 January 2003, and 31 December 2020, with at least one parent who was also reported to that agency during their childhood (*n* = 5861 children). The cohort was extracted using clinical administrative data, and logistic regression models were tested with the children’s CM types as the dependent variables. Homotypical continuity was found for: (1) physical abuse on the paternal side; (2) sexual abuse on the maternal side; and (3) exposure to domestic violence on the maternal side. Heterotypical continuity was also prevalent, but to a lesser extent. Interventions helping maltreated parents overcome their traumatic past are essential to foster intergenerational resilience.

## 1. Introduction

According to the World Health Organization, “child maltreatment (CM) is the abuse and neglect that occurs to children under 18 years of age […] which results in actual or potential harm to the child’s health, survival, development, or dignity in the context of a relationship of responsibility, trust, or power” [1]. CM is a highly prevalent public health problem, which can be emphasized based on the latest Canada-wide Incidence Study on Reported Child Abuse and Neglect that highlights an incidence rate of 16.5 per 1000 children [2]. CM has devastating consequences including suicidality, mortality and morbidity, psychopathology, substance misuse, risky behaviours, poor academic outcomes, and delinquent and criminal behaviour that can persist into adulthood [3,4,5]. These long-lasting consequences may be due to the well-documented association between child abuse and alterations in the brain structure and function and their related poorer treatment responses [6,7]. These long-term impacts of CM may lead to its intergenerational continuity (for reviews, see [8,9,10]), which refers to situations where both a child and at least one of their parents has been subjected to CM. While the study of the intergenerational continuity of CM has gained momentum in recent years [11], the following methodological limitations highlighted by Thornberry and colleagues [12] still plague this literature: unrepresentative samples, low participation rates and high attrition, use of retrospective data, same assessors for parents’ and children’s CM, unvalidated/unreliable measures of CM, unclear definitions of CM, and lack of long-term follow-ups [8]. Furthermore, research is still lacking in the examination of continuity on the paternal side, and the form intergenerational continuity takes for different CM types (e.g., neglect, exposure to domestic violence, psychological maltreatment). The current study will fill many of these gaps by examining the nature of intergenerational continuity of substantiated CM on the maternal and paternal side, while using population-level data from a child protection database that was collected over more than 40 years. 

Berzenski et al. [13] defined the homotypical form of continuity as both the child and the parent experiencing the same type(s) of CM, and heterotypical continuity as situations where the child and the parent experienced distinct types of CM. They argued for a multidimensional understanding of the intergenerational continuity of CM that distinguishes between these forms of continuity to better orient prevention initiatives. Similarly, they highlighted that while types of CM have different correlates and consequences, their continuity across generations may vary as well, supporting the need to move beyond the undifferentiated approach to CM that is commonly used in the literature where all types are combined into a general CM construct. Empirical findings support the simultaneous inclusion of CM types (versus a general CM score) within multivariate statistical models to account for a greater variance in developmental outcomes [14]. Similarly, the Dimensional Model of Adversity and Psychopathology proposed by McLaughlin and Sheridan [15] emphasised that threat (e.g., abuse) and deprivation (e.g., neglect) have differential impacts on functioning. Thus, different CM histories in parents may translate into different patterns of intergenerational continuity of CM due to distinct developmental outcomes. Unfortunately, many studies on the continuity of CM still use an undifferentiated perspective [8]. 

### 1.1. Homotypical Continuity

The available empirical literature tends to support the presence of homotypical continuity on the maternal side for several CM types [8,9,10], especially with physical abuse, which is the most explored CM type (e.g., [14,15,16,17,18]). For example, a German study [19] found that the use of physical violence was higher in parents with a history of minor and severe childhood physical abuse (30.8% and 38.8%, respectively) than in parents without such histories (11.3%). For child sexual abuse, homotypical continuity is also apparent. Specifically, studies documented that between 26.6% to 51.0% of children of sexually abused mothers were also subjected to sexual abuse [20,21,22,23]. Fewer studies have documented the continuity of neglect, psychological maltreatment, and exposure to domestic violence. Kim [17] highlighted that 21.1% of neglected parents used neglectful parenting with their own children compared to 9.3% for non-neglected parents. Cannon et al. [24] found that 49.1% of children whose mothers witnessed intimate partner violence in childhood also witnessed such violence. Finally, a study from Croatia showed that around 87% of parents with a history of psychological maltreatment reported psychologically maltreating their children [25]. While these studies provide support for the presence of the homotypical continuity of CM, particularly in mothers, a major limitation is that most of them only documented one or a few types of CM, and did not control for the presence of other forms of CM. Thus, due to the prevalence of polyvictimisation (exposure to more than one form of victimisation) [26,27,28], this approach may lead to an overestimation of homotypical continuity.

### 1.2. Heterotypical Continuity

Few studies examined the presence of the heterotypical continuity of CM. In Madigan and colleagues’ meta-analysis [9], significant continuity was identified from parental neglect to their offspring’s physical abuse or sexual abuse, but not to psychological maltreatment. Continuity starting with parental childhood physical abuse was also found with offspring’s experiences of neglect, sexual abuse, and psychological maltreatment. Continuity starting with parental childhood psychological maltreatment was identified with offspring’s physical and sexual abuse. Finally, parental childhood sexual abuse was significantly related to all three CM types for their offspring. Thus, current evidence, mostly based on studies with mothers, has shown that heterotypical continuity of CM may be present, especially with parents’ histories of physical or sexual abuse. Unfortunately, exposure to domestic violence is excluded from many studies, including Madigan and colleagues’ meta-analysis [9]. More studies are needed to examine the presence of both heterotypical and homotypical continuity for all CM types using multivariate statistical models and including fathers. 

### 1.3. Current Study

This study follows Berzenski and colleagues’ recommendation [13] to better delineate the phenomenon of the intergenerational continuity of CM. Our methodological design meets or exceeds 8 out of the 11 criteria proposed by Thornberry and colleagues [12] for intergenerational CM studies by using population-level administrative data with no missing data or attrition, including: control variables and all CM types; having different CM reporters for each generation; covering an extended period of exposure; and using valid and reliable CM assessments following clear legal definitions. Specifically, the current population-level study aimed to examine the presence of homotypical and heterotypical intergenerational continuity of substantiated CM on the maternal and paternal sides. Multivariate models were tested to determine whether once all parents’ CM types are simultaneously considered, one type is associated more strongly than the others with children’s CM, and whether these patterns are more reflective of homotypical or heterotypical continuity. Based on past empirical findings, and in line with Berzenski and colleagues [13], we hypothesized that homotypical continuity would be more prevalent than heterotypical continuity. 

## 2. Materials and Methods

### 2.1. Local Child Protection Services and Clinical Administrative Database 

In the Province of Quebec, the Youth Protection Act covers various situations where the security or development of children is considered to be in danger [29]. These include abandonment, neglect (physical, health-related, education-related) and serious risk of neglect, physical abuse and serious risk of physical abuse, sexual abuse and serious risk of sexual abuse, exposure to domestic violence, psychological maltreatment, and serious behaviour disturbances. All child protection trajectories begin with a report being made to the Director of Youth Protection by a concerned member of the public (e.g., family member, neighbour, family friend) or a professional (e.g., physician, teacher, psychologist) for one or several suspected situations that may compromise the security or development of a child. Once the report is received, a decision to retain the report for evaluation or not is made by child protection workers based on a preliminary assessment of the risks to the child’s security and development. If the report is accepted, the ensuing evaluation process considers several factors, including the nature, gravity, persistence, and frequency of facts in the report, child’s age and personal characteristics, ability and willingness of parents to correct the situation, and resources available around the child and parents. As soon as a report comes in, it is recorded using scroll-down menus in the child protection clinical-administrative database of the relevant child protection agency, and every additional decision is recorded as well. This information regarding a child’s protection services involvement (e.g., original reasons for reporting, decision to retain the report or not, conclusions of the evaluation regarding the presence of CM and the type of substantiated CM) is recorded by child protection workers involved with the case.

### 2.2. Study Cohort

The population-level cohort was extracted by a specialised analyst from the child protection clinical administrative database of the Centre Jeunesse de Montréal (CCSMTL), the agency serving the francophone community of Montreal, Quebec. Children are the unit of analysis in the current study. All children reported to the CCSMTL between 1 January 2003, and 31 December 2020, who also had at least one parent reported to that agency in their childhood, were included (*N* = 7923 children). Of these 7923 children, 5278 were included because their mother was reported to CCSMTL in her childhood, 1562 were included because their father was reported, and 1083 because both of their parents were reported in their childhood. The mothers had a mean of 2.43 children (*SD* = 1.48) in the cohort; for the fathers, it was 2.07 (*SD* = 1.34). Sociodemographic and child protection services (CPS) involvement characteristics for the cohort are presented in Table 1. In the current study, only substantiated reports were considered, leaving 5861 children, 5309 mothers, and 2208 fathers for the main analyses. The majority of children in this cohort (63.8%) were maltreated by both of their parents.

### 2.3. Variables

All variables used in this study were extracted from the clinical administrative database of the CCSMTL. Thus, they reflect information collected and recorded in real time by child protection workers involved with the children in this cohort and their parents at any moment of their CPS involvement.

#### Parents’ and Children’s Substantiated Child Maltreatment

Lifetime occurrences of substantiated neglect, physical abuse, sexual abuse, exposure to domestic violence, psychological maltreatment, and serious risk of abuse or neglect for both generations were dummy coded in the dataset (0 = absence, 1 = presence). These encompass all substantiated maltreatment types for an individual at any moment in their involvement (s) with the CCSMTL, so between the ages of 0 and 17 years old for parents, and between the ages of 0 and 17 or from 0 to the age at data extraction for children. As presented in Table 1, a variable documenting the presence of co-occurrent grounds for CPS involvement, including abandonment and serious behaviour disturbance in addition to maltreatment-related grounds, was dummy coded in the dataset (0 = single ground, 1 = co-occurrent grounds). Similarly, individuals with a history of at least one lifetime placement were identified using a dummy coded variable (0 = absence of placement, 1 = presence of placement).

#### Covariates

The sociodemographic characteristics were extracted from the clinical administrative database including child sex, child and parents’ age at first CCSMTL report, and parents’ age at the birth of their first reported child. A continuous variable was created to document the number of children each parent has in the present cohort. Finally, we extracted the immigration status of the children as an indicator of the cohort’s diversity (Table 1), since the ethno-racial information of the children was not reliably recorded in the database.

In addition to the five grounds related to CM, the presence of abandonment or serious behaviour disturbances as a lifetime substantiated grounds for CPS involvement in the parents’ childhood was included as a covariate in the multivariate models. These were dummy coded in the same way as the CM categories. These grounds were treated differently from the others as they do not constitute CM per se.

### 2.4. Analysis Plan

To ensure the absence of linearity among the covariates and determine the variance inflation factor (VIF) estimates, two linear regressions (fathers and mothers) were conducted with SPSS with the same covariates as the final regression models. This approach allowed us to determine the amount of variance of corresponding covariates that was increased due to multicollinearity. Tolerance was also examined. While there is no formal cut-off value when using VIF, a value exceeding 10 is considered indicative of multicollinearity [30]; for tolerance, values below 0.40 suggest concerns and values below 0.20 suggest serious multicollinearity in a model [31,32]. The tolerance values were all above 0.67 and the VIF statistics were all below 2, reflecting no concerns of multicollinearity. Then, a series of logistic regression models was tested (separately for mother–child and father–child dyads) with children CM types as the dependent variables. Relevant sociodemographic and CM variables were entered including child sex, child age at first report to CCSMTL, parents’ age at birth of the target child, parents’ histories of substantiated CM types, and parents’ substantiated abandonment, and serious behaviour disturbance histories. For binary categorical variables, the category of reference was set to be 0 = absence; for sex specifically, the category of reference was being a girl. 

## 3. Results

### 3.1. Logistic Regressions Predicting Children Maltreatment Types

Table 2 and Table 3 display the detailed findings of the regression models with the mothers and fathers, respectively.

### 3.2. Neglect

The logistic regression with the mothers was statistically significant. The model showed that some sociodemographic characteristics were associated with the risk of offspring’s substantiated neglect: older children and children of younger mothers were more likely to be substantiated for neglect. CPS-involved children of mothers with a history of substantiated neglect or psychological maltreatment were less likely to be substantiated for neglect.

The logistic regression with the fathers was also statistically significant. Children with younger fathers were more at risk, while a history of paternal substantiated neglect or psychological maltreatment was associated with a lower risk of substantiation for neglect. On the other hand, having a father with substantiated abandonment doubled the risk for the offspring to be substantiated for neglect.

### 3.3. Physical Abuse

The regression with the mothers was statistically significant. The model showed that children who were older or boys were more likely to be substantiated for physical abuse than younger children and girls; children with younger mothers were also at increased risk. Histories of neglect, child sexual abuse, psychological maltreatment, or behaviour problems on the maternal side were associated with a lower risk of being substantiated for physical abuse.

The regression with the fathers was also statistically significant. Boys and older children had a greater risk of being substantiated for physical abuse. A paternal history of physical abuse was associated with a greater risk of offspring being substantiated for physical abuse as well.

### 3.4. Sexual Abuse

The regression with the mothers was statistically significant. Older children with younger mothers were at increased risk of substantiated sexual abuse; girls were three times more likely to be substantiated for sexual abuse than boys. A history of maternal neglect was associated with a lower risk of substantiation for sexual abuse, while a history of sexual abuse increased the risk by 1.42 times.

The model was also statistically significant with the fathers. The results showed an increased risk of substantiation for sexual abuse for older children and girls. A history of paternal physical abuse was associated with a lower risk of being substantiated for sexual abuse, while it was the reverse for a history of abandonment, which increased this risk by 2.74 times.

### 3.5. Exposure to Domestic Violence

The regression with the mothers was statistically significant. The model showed that younger children were at increased risk of being substantiated for exposure to domestic violence. In terms of maternal factors, a history of physical abuse or exposure to domestic violence was related to an increased risk of offspring substantiation for exposure to domestic violence, which reflected both heterotypical and homotypical trajectories; the opposite was true for a maternal history of serious risk of abuse.

The model with the fathers was also statistically significant. Younger children were at increased risk of substantiation for exposure to domestic violence in the complete model with the fathers. A history of abandonment on the father’s side was associated with a lower risk of substantiation for exposure to domestic violence in children.

### 3.6. Psychological Maltreatment

The overall regression with the mothers was not statistically significant, but the one with the fathers was. The only statistically significant association was a negative one with paternal abandonment.

### 3.7. Serious Risk of Neglect

The logistic regression with the mothers was statistically significant. Younger children and children with younger mothers had an increased risk of being substantiated for serious risk of neglect. While a history of maternal neglect or physical abuse was related to lower odds of offspring substantiation for serious risk of neglect, it was the reverse when considering a history of sexual abuse or behaviour problems.

The model was also statistically significant with fathers and yielded the same findings as the model with mothers regarding the age of the offspring and fathers. A paternal history of abandonment was related to a lower risk of offspring substantiation for serious risk of neglect.

### 3.8. Serious Risk of Abuse

The logistic regression with the mothers was statistically significant. Younger children had an increased risk of being substantiated for serious risk of abuse. A maternal history of exposure to domestic violence was negatively related to substantiation for the risk of abuse.

Similarly, the regression with the fathers was also statistically significant. The model with fathers showed that younger children were at increased risk of substantiation for risk of abuse than older ones.

### 3.9. Summary of Key Findings

The positive associations identified with the maternal histories of CM involved mothers’ exposure to domestic violence with children’s exposure to domestic violence; maternal physical abuse was associated with children’s exposure to domestic violence. A positive association was found between a maternal history of sexual abuse and behaviour disturbances, and children’s substantiation for serious risk of neglect. Sexual abuse on the maternal side was associated with a higher risk of children’s substantiation for sexual abuse. On the paternal side, a history of abandonment was related to a higher risk of children’s neglect and sexual abuse substantiation, and a history of serious risk of abuse was related to a higher risk of children’s psychological maltreatment substantiation. A paternal history of physical abuse increased the risk of substantiation for physical abuse in children. When parents’ neglect emerged as a significant factor, it was always negatively associated with children’s CM.

## 4. Discussion

In Berzenski and colleagues’ chapter [13], they argued for a multidimensional understanding of the intergenerational continuity of CM that would better inform prevention and intervention practices. The aim of this study was to examine the homotypical and heterotypical forms of intergenerational continuity of CM, on maternal and paternal sides, in a population cohort of families using multivariate models. In line with past empirical findings [17,19], relatively more homotypical than heterotypical forms of continuity were identified in our analyses. Specifically, three homotypical forms of continuity (out of a potential of 14; 21.4%) were confirmed: (1) physical abuse on the paternal side; (2) sexual abuse on the maternal side; and (3) exposure to domestic violence on the maternal side. Comparatively, only 7.1% of the possible heterotypical forms emerged as significant. Theoretical perspectives, such as social learning [33], life-course [34], and attachment [35] theories, postulate that parents reproduce the parenting models they were exposed to with their own children, or may be more accepting of the use of similar practices from their co-parent [36]. Accordingly, this would naturally lead to greater odds of homotypical than heterotypical forms of continuity of intergenerational CM. All in all, distinguishing continuity forms is clinically relevant to inform prevention initiatives. Future studies aiming to identify mechanisms involved in the intergenerational continuity of CM should examine both forms of continuity and consider all CM types.

### 4.1. Homotypical Continuity of Sexual and Physical Abuse

Physical and sexual abuse are the only CM types that had gendered patterns in this cohort, with boys being more likely to be substantiated for physical abuse and girls being more likely to be substantiated for sexual abuse. Interestingly, these CM types also showed gendered patterns of continuity with homotypical continuity forms identified for physical abuse, but only on the paternal side, and for sexual abuse, but only on the maternal side. Our findings pertaining to sexual abuse are congruent with those of Madigan and colleagues [9] that identified increased probabilities of CSA intergenerational continuity in studies with higher percentages of girls versus boys and of mothers versus fathers. However, this meta-analysis did not find a gendered pattern for the continuity of physical abuse. Findings pertaining to the continuity of sexual abuse through the maternal side might be reflective of the low count of fathers with a history of sexual abuse in this cohort. The current study does not allow the identification of potential mechanisms underlying these gendered patterns, but our results highlight the importance of reliably including fathers in studies on intergenerational maltreatment. Future studies should aim to replicate our findings and to identify potential explanations.

### 4.2. Exposure to Domestic Violence: Homotypical and Heterotypical Continuity

In our cohort, homotypical continuity on the maternal side was identified with exposure to domestic violence, and heterotypical continuity from mothers’ physical abuse to children’s exposure to domestic violence emerged. While both men and women can be the victim of domestic violence, women are still the victims of more severe and chronic forms of domestic violence; they are disproportionately represented in legal cases of domestic violence compared to men [37,38,39]. Therefore, in our cohort of families involved with CPS, these homotypical and heterotypical continuities likely represent victim-to-victim cycles of CM wherein the parent maltreated in childhood is later revictimised in the context of an intimate relationship, leading to the offspring’s exposure to violence. Ample literature has shown that women exposed to domestic violence in childhood are more likely to be the victim of intimate partner violence in adolescence and adulthood [40,41]. There is also considerable empirical support demonstrating that childhood physical abuse is a risk factor for later becoming the victim of domestic violence, especially for women [42,43]. These revictimisation patterns are consistent with our findings that children are more likely to be substantiated for exposure to domestic violence if their mother has a history of physical abuse or exposure to domestic violence.

### 4.3. Abandonment in Fathers

A history of abandonment on the paternal side was significant in almost all second-generation CM types and was influential in predicting increased risks of sexual abuse and neglect. As reported by the consulted field workers from CPS, abandonment is rarely a primary or unique ground for CPS involvement, and often arises later when the children’s situation has deteriorated. Similarly, in our sample, only 33.1% of fathers with substantiated abandonment had only one CPS ground in their file compared to 74.1% of fathers without abandonment. The more severe presentation of cases of abandonment might partly explain why this ground was significant for many offspring’s CM types.

Specifically, in the Youth Protection Act, abandonment “refers to a situation in which a child’s parents are deceased or fail to provide for the child’s care, maintenance or education and those responsibilities are not assumed by another person in accordance with the child’s needs” [29]. Based on this definition and apart from situations where both parents are deceased, abandonment could be construed as an extreme form of neglect that fathers were likely to reproduce with their own offspring, representing a continuity analogous to homotypical ones. Regarding the increased risk of substantiation for sexual abuse, we are unfortunately limited in our ability to interpret this finding given that our dataset does not identify the perpetrator of the sexual abuse. When the father is not the perpetrator of the abuse, this may represent a victim-to-victim cycle of CM where the parent maltreated in childhood did not maltreat their child, but did fail to protect them against a sexual predator in the environment. In this case, a possible explanation is that abandoned fathers are less likely to have grown up with positive models of parenting that were able to offer appropriate protection to their children, leading to a reduced capacity to do so when becoming a parent. In cases where the perpetrator of the sexual abuse is the father, we might propose that abandonment in childhood led to the development of personal and relational characteristics renowned as risk factors for becoming a child sexual abuse perpetrator (e.g., social skills deficits, powerlessness, low self-esteem, feelings of humiliation, insecure attachment, discomfort and dissatisfaction in relationships with adults, loneliness) [44]. The significant role of a father’s history of abandonment in our cohort, a clearly understudied form of childhood adversity, highlight the need for further exploration of this phenomenon.

### 4.4. Neglect as a Unique CM Type

Interestingly, neglect appears to be distinct from other CM types as maternal and paternal histories of neglect were negatively associated with the risks of offspring’s substantiation for neglect, and maternal histories of neglect were associated with lower risks of substantiation for physical abuse, sexual abuse, and serious risk of neglect. Other associations were not significant. Because all children and parents in our cohort have a history of substantiated CM, this finding does not mean that parents with a history of neglect are less likely to have children that will be maltreated. However, it indicates that a such history might not be helpful in identifying children at risk for specific CM types; it might be a non-specific risk factor in terms of second-generation CM. The distinguishing feature of neglect, compared to other CM types, is that deprivation is at its core instead of harm or threat of harm, leading to different consequences in terms of development [45]. Specifically, neglected children are deprived of a sensitive and responsive caregiver, resulting in major reductions in the stimulation received [45]. Thus, neglected children show deficits in many areas including affective functioning (e.g., disorganised and insecure attachment, emotion regulation) and cognitive development (e.g., cognitive ability, associative learning, language, executive functioning). These extensive developmental implications may increase the risk of second-generation CM in many ways and without it being specific to one CM type. Relatedly, chronic difficult life conditions (e.g., poverty, addiction) are often present in families where there is neglect. The intergenerational transmission of these life conditions, which are risk factors for all CM types [45,46], might also explain why parental neglect appears as a diffuse risk for offspring CM.

Furthermore, serious risk of neglect was the CM type for offspring with the highest proportion of variance explained by our study variables (19.8%), far beyond those of other CM types (0.5–9.5%). The serious risk of neglect category is used by CPS when there is a risk or sufficient concern that a caregiver is not providing for the child’s basic physical, health, and educational needs without sufficient proof for neglect substantiation [29]. Families substantiated for serious risk of neglect are often characterised by risky lifestyles (e.g., problematic substance use, criminal activities), deprived or risky environments (e.g., extreme poverty, unsafe environment), or caregivers’ physical and mental health problems (e.g., intellectual disability, immaturity/impulsivity). In this context, it stands to reason that younger parents and mothers with a history of serious behaviour disturbances are more likely to have their children substantiated for serious risk of neglect. The well-documented and long-lasting consequences of child sexual abuse on functioning could also explain the positive association between a history of child sexual abuse in mothers and offspring substantiation for serious risk of neglect [47]. While other CM types are also associated with similar consequences in adulthood, it appears that child sexual abuse exerts a stronger influence on the risk of neglect when all CM types are covaried in one statistical model. These results could also be indicative of biases in CPS where parents with specific histories of CM (i.e., sexual abuse) and behaviour disturbances are more likely to be flagged for serious risk of neglect—so with no conclusive evidence of actual neglect—than those without such characteristics [48]. Further investigations are required to provide a more in-depth understanding of these matters.

### 4.5. Limitations

This study filled some gaps in the current literature regarding the intergenerational continuity of CM by examining homotypical and heterotypical continuity using multivariate models in a populational cohort of families involved with CPS. Nevertheless, some limitations should be acknowledged. The use of clinical administrative databases, while highly cost-effective and providing valid, population-level, and prospective data on reported CM, limits the number of clinical details that are available. They also only account for the CM cases that are officially reported to the authorities, so they are not representative of all CM cases. The study of the intergenerational continuity of CM using clinical administrative data also required going back many decades in the system. In this context, acknowledgement of historical changes in legislation related to CM is needed. It is also worth mentioning that some CM types are less represented in the current study (e.g., abandonment, paternal sexual abuse and exposure to domestic violence) and that our cohort of fathers was smaller, limiting our statistical power. Another important limitation is that our study could not include discontinuity groups for comparison, such as parents with a history of substantiated CM with non-maltreated children (CM discontinuity), and parents without a history of CM with CPS-involved children (no CM discontinuity).

### 4.6. Implications for Research and Practice

Future studies should replicate our findings and expand on them by including comparison groups and additional factors that might explain the mechanisms underlying the homotypical and heterotypical continuity of CM types. As the explained variances of CM in our study were generally small, other factors should be considered to gain a more comprehensive understanding as to why intergenerational CM might take one form instead of another in impacted families. For instance, future studies could examine the role of paternal abandonment on the next generation, and the effects of parental neglect versus abuse on the second generation’s CM status. As the intergenerational continuity of CM is now a well-documented phenomenon [9], a multidimensional and in-depth understanding is required to improve our ability to intervene to prevent these cycles from occurring, and, as a whole, prevent CM from occurring [13]. Our findings show that patterns of continuity differ, but are present on both paternal and maternal sides, emphasizing the need for a systematic inclusion of fathers in future studies, and for a family-system approach to intervention and prevention with at-risk families. The greater presence of homotypical continuity emphasizes the need for interventions helping parents with a CM history to break free of these deterministic experiences and develop more positive models of parenting and appropriate parenting practices. This might be particularly relevant for younger parents, as parents’ age was identified as a risk factor for many CM types.

While recognising the role of bias when examining families with generational CPS involvement and the collective goal to prevent CM before it occurs (primary prevention), experts in the field also stress the importance of simultaneously implementing secondary and tertiary prevention interventions to limit the continuity of CM across generations [49]. Secondary prevention requires implementing interventions to prevent CM in at-risk populations, and tertiary prevention focuses on working with families where CM occurred to limit its negative sequelae and prevent its recurrence [50]. Specifically, if the replication of our findings related to the homotypical continuity of sexual and physical abuse is conclusive, secondary and tertiary prevention should target father–boy dyads for physical abuse, and to mother–girl dyads for sexual abuse. Regarding our findings for exposure to domestic violence, it appears essential to help children and women exposed to domestic violence and physical abuse in childhood to process their trauma and recover a resilient developmental trajectory to foster the discontinuity of CM. Overall, providing early interventions to children who were maltreated is desirable to prevent the long-term deleterious consequences of early adversity and their spill-over effect on the next generations.

## 5. Conclusions

To conclude, the current population-level study aimed to examine the presence of homotypical and heterotypical intergenerational continuity of substantiated CM on the maternal and paternal sides. Our findings demonstrated a greater presence of homotypical continuity (i.e., physical abuse on the paternal side; sexual abuse and exposure to domestic violence on the maternal side), but heterotypical continuity (e.g., physical abuse to exposure to domestic violence on the maternal side) also prevailed. Furthermore, the findings underline that both parental histories should be considered when screening CM in children and that treatment measures such as childhood trauma processing and parenting skills training should be offered within a family-systems perspective. More studies are needed to deepen our understanding of the mechanisms underlying specific patterns of intergenerational CM. Documenting family patterns of CM and child welfare involvement allows for transparency and accountability, as well as informed decision making to limit CM in our communities.

## Figures and Tables

**Table 1:** Characteristics of the cohort.

Variable	*M* (*SD*)/*n* (%)		
	Children	Mothers	Fathers
Age at birth of 1st reported child	N/A	20.25 (3.82)	23.16 (4.62)
Age at first CCSMTL report	3.20 (3.77)	11.52 (4.41)	11.19 (4.37)
Sex		N/A	N/A
Boys	4130 (52.1%)		
Girls	3790 (47.8%)		
Immigration status		N/A	N/A
1st or 2nd generation	2174 (27.4%)		
Lifetime presence of substantiated co-occurrence	4113 (70.2%)	1927 (36.3%)	641 (29.1%)
Lifetime substantiated neglect	2900 (49.5%)	778 (14.7%)	199 (9.0%)
Lifetime substantiated physical abuse	1044 (17.8%)	931 (14.6%)	317 (14.4%)
Lifetime substantiated sexual abuse	340 (5.8%)	863 (16.3%)	104 (4.7%)
Lifetime substantiated exposure to domestic violence	2158 (36.8%)	301 (5.7%)	81 (3.7%)
Lifetime substantiated psychological maltreatment	2448 (41.8%)	504 (9.5%)	146 (6.6%)
Lifetime substantiated serious risk of abuse or neglect	3845 (65.6%)	2209 (41.6%)	860 (39.0%)
Lifetime substantiated abandonment	151 (2.6%)	329 (6.2%)	133 (6.0%)
Lifetime substantiated behavioural disturbance	550 (9.4%)	2598 (48.9%)	1356 (61.5%)
No substantiation	2062 (26.0%)	1052 (16.5%)	438 (16.6%)
Presence of at least one placement	2461 (31.1%)	2700 (42.4%)	1099 (41.5%)

**Table 2:** Mothers’ child maltreatment histories predicting child maltreatment in offspring.

	Children Neglect		Children Physical Abuse		Children Sexual Abuse		Children Exposure to DV		Children Psych. Maltreatment		Children Serious Risk of Neglect		Children Serious Risk of Abuse	
	*B (SE)*	OR	*B (SE)*	OR	*B (SE)*	OR	*B (SE)*	OR	*B (SE)*	OR	*B (SE)*	OR	*B (SE)*	OR
Child sex			0.22 (0.08) **	1.24	−1.13 (0.15) ***	0.32								
Age—child	0.02 (0.01) *	1.02	0.05 (0.01) ***	1.05	0.08 (0.02) ***	1.0	−0.05 (0.01) ***	0.95			−0.26 (0.01) ***	0.77	−0.08 (0.02) ***	0.92
Age—mother	−0.04 (0.01) ***	0.96	−0.05 (0.01) ***	0.95	−0.05 (0.02) **	0.95					−0.03 (0.01) ***	0.97		
Neglect	−0.31 (0.10) **	0.73	−0.29 (0.15) *	0.75	−0.69 (0.28) **	0.50					−0.38 (0.11) ***	0.68		
Physical abuse							0.27 (0.09) **	1.31			−0.40 (0.10) ***	0.67		
Sexual abuse			−0.37 (0.13) **	0.69	0.35 (0.18) *	1.42					0.27 (0.10) ***	1.31		
Behaviour disturbances			−0.35 (0.10) ***	0.71							0.19 (0.08) *	1.20		
Exposure to domestic violence							0.43 (0.16) **	1.54	0.40 (0.16) **	1.49			−0.52 (0.25) *	0.59
Psychological maltreatment	−0.30 (0.12) *	0.74	−0.41 (0.17) *	0.67										
Serious risk of neglect														
Serious risk of abuse							−0.56 (0.24) *	0.57						
Abandonment														
*R*^2^ (Nagelkerke)	2.50%		3.80%		9.50%		1.80%		0.50%		1.80%		1.80%	
*χ*^2^ (12, *N* = 4066)	77.15 ***		94.76 ***		147.52 ***		52.28 ***		14.83		639.28 ***		39.72 ***	
% Correct classification	55.00%		82.40%		93.70%		64.40%		57.80%		70.60%		87.00%	

Note. * *p* < 0.05; ** *p* < 0.01; *** *p* < 0.001. Only statistically significant findings are presented.

**Table 3:** Fathers’ child maltreatment histories predicting child maltreatment in offspring.

	Children Neglect		Children Physical Abuse		Children Sexual Abuse		Children Exposure to DV		Children Psych. Maltreatment		Children Serious Risk of Neglect		Children Serious Risk of Abuse	
	*B (SE)*	OR	*B (SE)*	OR	*B (SE)*	OR	*B (SE)*	OR	*B (SE)*	OR	*B (SE)*	OR	*B (SE)*	OR
Child sex			0.28 (0.14) *	1.33	−1.09 (0.23) ***	0.34								
Age—child			0.05 (0.02) **	1.05	0.06 (0.03) *	1.06	−0.07 (0.02) ***	0.93			−0.28 (0.02) ***	0.76	−0.13 (0.03) ***	0.88
Age—father	−0.05 (0.01) ***	0.95	−0.03 (0.01) *	0.97							−0.03 (0.01) *	0.97		
Neglect	−0.47 (0.20) **	0.63												
Physical abuse			0.45 (0.18) **	1.56	−1.53 (0.53) **	0.22								
Sexual abuse														
Behaviour disturbances														
Exposure to domestic violence														
Psychological maltreatment	−0.61 (0.24) **	0.55												
Serious risk of neglect														
Serious risk of abuse														
Abandonment	0.69 (0.22) **	2.00			1.01 (0.34) **	2.74	−0.62 (0.23) **	0.54	−0.87 (0.24) ***	0.42	−0.48 (0.23) *	0.62		
*R*^2^ (Nagelkerke)	3.90%		3.10%		11.00%		3.90%		2.20%		22.90%		3.80%	
*χ*^2^ (12, *N* = 1634)	48.79 ***		30.61 **		67.04 ***		47.35 ***		27.25 **		303.73 ***		33.59 ***	
% Correct classification	56.40%		83.40%		94.00%		58.90%		57.40%		70.10%		86.90%	

Note. * *p* < 0.05; ** *p* < 0.01; *** *p* < 0.001. Only statistically significant findings are presented.

## Data Availability

We do not have permission to share this research data.
